# Neutrophil extracellular traps promote cancer-associated inflammation and myocardial stress

**DOI:** 10.1080/2162402X.2022.2049487

**Published:** 2022-03-14

**Authors:** J. Cedervall, M. Herre, A. Dragomir, F. Rabelo-Melo, A. Svensson, C. Thålin, A. Rosell, V. Hjalmar, H. Wallén, H. Lindman, G. Pejler, E. Hagström, M. Hultström, A. Larsson, AK. Olsson

**Affiliations:** a Department of Medical Biochemistry and Microbiology, Science for Life Laboratory, Uppsala University, Biomedical Center, Uppsala, Sweden; b Department of Immunology, Genetics and Pathology, Uppsala University, Rudbeck Laboratory, Uppsala, Sweden; c Department of Medical Cell Biology, Integrative Physiology, Uppsala University, Uppsala, Sweden; d Department of Surgical Sciences, Anaesthesiology and Intensive Care Medicine, Uppsala University, Uppsala, Sweden; e Department of Clinical Sciences, Danderyd Hospital, Karolinska Institutet, Stockholm, Sweden; f Diagnostic Centre, Danderyd Hospital, Karolinska Institute, Stockholm, Sweden; g Department of Medical Sciences, Uppsala University, Uppsala, Sweden

**Keywords:** Neutrophil extracellular traps, NETs, cancer, cardiac, hypertrophy, inflammation

## Abstract

Cancer is associated with systemic pathologies that contribute to mortality, such as thrombosis and distant organ failure. The aim of this study was to investigate the potential role of neutrophil extracellular traps (NETs) in myocardial inflammation and tissue damage in treatment-naïve individuals with cancer. Mice with mammary carcinoma (MMTV-PyMT) had increased plasma levels of NETs measured as H3Cit-DNA complexes, paralleled with elevated coagulation, compared to healthy littermates. MMTV-PyMT mice displayed upregulation of pro-inflammatory markers in the heart, myocardial hypertrophy and elevated cardiac disease biomarkers in the blood, but not echocardiographic heart failure. Moreover, increased endothelial proliferation was observed in hearts from tumor-bearing mice. Removal of NETs by DNase I treatment suppressed the myocardial inflammation, expression of cardiac disease biomarkers and endothelial proliferation. Compared to a healthy control group, treatment-naïve cancer patients with different malignant disorders had increased NET formation, which correlated to plasma levels of the inflammatory marker CRP and the cardiac disease biomarkers NT-proBNP and sTNFR1, in agreement with the mouse data. Altogether, our data indicate that NETs contribute to inflammation and myocardial stress during malignancy. These findings suggest NETs as potential therapeutic targets to prevent cardiac inflammation and dysfunction in cancer patients.

## Introduction

Cancer is a complex disease that also affects organs that are not direct sites for primary or secondary tumor growth. Cancer patients are frequently affected by impaired function or even failure of organs, despite the absence of tumor growth at these particular sites. Approximately 50% of all patients with a malignant disease, regardless of type, have impaired renal function already at the time of diagnosis.^1–3^ Since a majority of the chemotherapeutics currently in clinical use are excreted via the kidneys, a suboptimal renal function requires dose reductions to avoid cytotoxic effects on the kidneys, which would otherwise accelerate the renal injury even further. As a consequence, the possibility of cure from the malignant disease is reduced.

Likewise, pathological effects have been observed in hearts from cancer patients. Already in the 1960s, it was demonstrated that hearts from cancer patients were of reduced weight and had thinner ventricular walls than hearts from healthy individuals.^4^ More recently, it was reported that cancer patients have elevated expression of biomarkers for cardiac disease^5^ and myocardial fibrosis has been observed in histopathological analysis of hearts from cancer patients.^6^ Impaired cardiac function in cancer patients is a common side-effect of chemotherapy. However, signs of heart dysfunction have also been observed even before initiation of treatment, indicating that the impaired function can also be caused by the malignancy itself.^5,7^ In a preclinical study using subcutaneously grafted Lewis lung carcinoma (LLC), innervation of the myocardium was impaired and the mice had elevated plasma levels of IL-1β, indicative of inflammation.^8^

It is well established that cancer patients are at increased risk for thrombosis.^9,10^ There are several reasons, for example tumor-associated expression of tissue factor (TF), as well as poor vascular integrity exposing subendothelial pro-thrombotic factors such as collagen.^11^ Accumulating data also show that the immune system can promote thrombosis by formation of neutrophil extracellular traps (NETs). NET formation was first described as a defense mechanism to fight severe bacterial infections,^12^ but has now also been connected to other infectious diseases, as well as conditions characterized by sterile inflammation, such as rheumatoid arthritis, diabetes and cancer.^13^ NETs are formed by activated neutrophils expelling decondensed chromatin, decorated with granular proteases such as neutrophil elastase (NE) and myeloperoxidase (MPO), resulting in a web-like structure. The presence of extracellular DNA and histones, potent platelet activators, and the fact that NETs can activate factor XII,^14^ inducer of the intrinsic coagulation pathway, renders the NETs pro-thrombotic. In addition, TF, as well as TF-containing extracellular vesicles, have been found to interact with NETs,^15,16^ further adding to their thrombotic capacity. Due to the pro-thrombotic nature of NETs, their potential contribution to cancer-associated thrombosis (CAT) has gained much attention.^17,18^

In 2015, our research group identified tumor-induced NETs as a previously unknown cause of kidney vascular dysfunction in mice with cancer.^19^ Using mouse models for pancreatic insulinoma (RIP1-Tag2) and mammary carcinoma (MMTV-PyMT), we found that platelet-neutrophil complexes, indicative of NETs, were found exclusively in the glomerular vasculature of tumor-bearing mice, but never in healthy littermates. The renal vasculature showed decreased perfusion and increased leakiness, and the kidneys displayed an inflammatory state with expression of pro-inflammatory cytokines, infiltration of immune cells and endothelial activation in individuals with cancer.^19^ There were also signs of renal dysfunction, characterized by creatinine accumulation in the blood, proteinuria, and morphological alterations in the kidney glomeruli in tumor-bearing mice.^20^ Treatment with DNase I, a well-established method to resolve NETs, restored vessel function, suppressed the inflammation and improved renal function.^19,20^

We have also found reduced perfusion of the cardiac vasculature and enhanced numbers of neutrophils in the myocardium of tumor-bearing mice, indicating a systemic inflammatory state and not only restricted to the kidneys.^19^ NETs have indeed been associated with cardiovascular disease,^21^ but primarily in nonmalignant conditions. For example, NETs were shown to contribute to ischemia-reperfusion injury in a surgical mouse model for myocardial infarction.^22^ In patients, NETs have been associated with ischemic events such as acute ischemic stroke and myocardial infarction.^23,24^ NET formation was found to correlate with coagulation, as determined by quantification of thrombin-anti-thrombin (TAT) complexes, in patients with coronary artery disease.^23^ A connection between cancer, NETs and the cardiac biomarker high sensitive Troponin T (hsTnT) in patients with ischemic stroke was reported earlier.^25,26^ Based on these observations, we hypothesize that NETs and associated thrombi formation are potential drivers of cardiovascular injury in cancer. In the current study, we investigate whether cancer is a direct cause of cardiac injury, both in a mouse model of mammary carcinoma and in treatment-naïve patients with different malignancies. Moreover, we address the role of NETs in expression of biomarkers for cardiac function in cancer and the utility of NETs as therapeutic targets to counteract cancer-associated myocardial injury in mice.

## Material and methods

### Mice

Animal work was approved by the local ethics committee (dnr: C129/15;14613/2020) and performed according to the United Kingdom Coordinating Committee on Cancer Research guidelines for the welfare of animals in experimental neoplasia. MMTV-PyMT mice (genetic background FVB/n) were used as a model for mammary carcinoma in this study. The MMTV-PyMT mouse expresses the mouse polyoma middle-T antigen (PyMT) under the control of the mouse mammary tumor virus (MMTV) promotor.^27^ These mice uniformly develop adenocarcinomas of all mammary epithelia by 8–10 weeks of age, with a high incidence of pulmonary metastases. Tumor formation and progression in these mice is characterized by four stages: hyperplasia, adenoma/mammary intra-epithelial neoplasia, and early and late carcinoma.^28^ Other similarities to the human situation are the gradual loss of steroid hormone receptors and beta1-integrin, which is associated with overexpression of ErbB2 and cyclin D1.^29^ Mice were sacrificed and analyzed at the age of 14 weeks. Littermates lacking the PyMT transgene were used as healthy controls. Genotyping to detect the PyMT transgene was performed by PCR on DNA extracted from tail biopsies, using the following primer sequences:

5´-CGGCGGAGCGAGGAACTGAGGAGAG-3′,

5′-TCAGAAGACTCGGCAGTCTTAGGCG-3′.

### Human samples

Analysis of human samples complies with the Declaration of Helsinki and was approved by the Ethical Review Board in Stockholm (dnr 2020–00186; dnr 2017/2160-31/1; 2015/1533-31/1). Written informed consent was received from participants prior to inclusion in the study.

Citrated plasma from 33 patients diagnosed with different types of malignancies were included (Table S1). The patients were investigated at the Diagnostic Center (DC), Danderyd hospital, and had not initiated any treatment before the plasma was collected (Supplemental Table 1). Plasma samples from matched healthy individuals (control group) were provided from Danderyds Hospital, Karolinska Institute. Platelet-poor plasma was prepared from whole blood within 3 hours of sampling by centrifugation (2000 × g, 20 minutes, room temperature), and stored at −80°C. The study complies with the Declaration of Helsinki and was approved by the Ethical Review Board in Stockholm (dnr 2020–00186; dnr 2017/2160-31/1; 2015/1533-31/1).

### DNase I treatment

Mice were treated by daily intraperitoneal injections of DNase I (10 U in PBS, EN0521; ThermoFisher Scientific) for 3 days before sacrifice. Blood or organs were collected as described under respective analysis methods.

### Platelet depletion

Mice were intraperitoneally injected with anti-Gp1bα antibody (Emfret Analysis, R300) every third day for a total of 6 injections. The mice were injected with a dose of 4 μg antibody/g body weight at the first occasion and subsequently with 2 μg antibody/g body weight. Mice were sacrificed the day after the last injection. Blood was collected as described below.

### ELISA for NET detection in mouse and human plasma

Plasma was collected by terminal heart puncture from mice anaesthetized by intraperitoneal injection of 2% avertin, using citrate (0.011 M) as an anti-coagulant. Citrated venous plasma samples from cancer patients and healthy individuals were collected at Danderyd hospital. ELISA plates (F96 Maxisorp Nunc-immuno plate; Thermo Fisher) were coated with the H3Cit antibody (ab5103; abcam) diluted 1:200 for mouse and 1:500 for human samples or the MPO antibody (HM2164-clone 266–6K1; Hycult Biotechnology) (only human samples) diluted 1:20 and incubated overnight at 4°C. Wells were washed with PBS and blocked with 3% BSA for 1 hour at room temperature. After a washing step, 20 µl undiluted mouse or human plasma were added together with 80 µl or 30 µl dsDNA-peroxidase (POD) antibody (Cell Death ELISA^PLUS^; Roche) for the H3Cit and MPO ELISA, respectively, and incubated for 2 hours at room temperature. The DNA-POD antibody was diluted 1:20 for mouse, 1:100 for human H3Cit and 1:40 for human MPO detection. Following a washing step, TMB (T8665; Sigma) was added and the absorbance was measured at 650 nm with a microplate reader. Neutrophil Elastase was analyzed using a Human PMN Elastase ELISA kit (ab119553; Abcam), following instructions from the manufacturer.

### TAT assay

The TAT assay was performed on mouse plasma (collected as described above) using the Enzygnost TAT mikro kit (OWMGG15E33; Siemens), according to the manufacturer’s instruction.

### Analysis of biomarkers in mouse

ELISA was used to analyze the level of biomarkers for cardiovascular disease in the circulation. Serum was collected either from the tail vein, or by terminal heart puncture from mice anaesthetized by intraperitoneal injection of 2% avertin. GDF-15 was analyzed in mouse serum using the Mouse Growth/Differentiation factor 15 (GDF-15) SimpleStep ELISA kit (ab216947; Abcam), and soluble TNFR1 was analyzed in mouse serum using the Rat TNFR1 SimpleStep ELISA kit (ab231925; Abcam), according to the manufacturer’s instruction.

### Analysis of biomarkers in patient plasma

All biomarker assays were performed on citrated plasma. Platelet-poor plasma was prepared from whole blood within 3 hours of sampling by centrifugation (2000 × g, 20 minutes, room temperature) and stored at −80°C. Commercial sandwich ELISA was used to analyze the level of GDF-15 (DY957; R&D Systems), TNFR1 (DY225; R&D Systems) and TNFR2 (DY726; R&D Systems). Particle enhanced turbidimetric assay (PETIA) methodology was used to measure high sensitivity (hs) CRP. The reagents for CRP analysis were from Abbott Laboratories (Abbott Park, IL, USA), applied on a Mindray BS380 chemistry analyzer (Mindray, Shenzhen, China). NT-proBNP was analyzed using a Cobas 8000 (Roche Diagnostics, Mannheim, Germany). The instrument had a total coefficient of variation (CV) of 1.3% at 2060 ng/L and 0.9% at 107 ng/L. High sensitivity troponin I was measured on an Architect ci16200 (Abbott, Abbott Park, IL, USA). The instrument had a CV of 5.5% at 22 ng/L and 4.4% at 200 ng/L.

### Histology

Mice were anaestheitized with 4% isoflurane and hearts were dissected. Hearts were fixed in formalin buffer, embedded in paraffine and sectioned (5 μm) as per routine procedures for light microscopy. Tissue sections were stained with hematoxylin-eosin or Masson´s Trichrome, and analyzed by conventional light microscopy by a certified pathologist.

### Electron microscopy

For transmission electron microscopy (TEM), heart tissue from three control mice, three PyMT+ mice and three DNase-I treated PyMT+ mice were examined. Mice were anaesthetized with 4% isoflurane and dissected hearts were fixed in buffer containing 2.5% glutaraldehyde and 1% formalin at 4°C overnight. The tissue was embedded using the Eponate 12 Embedding kit (Ted Pella Inc., USA), and sections of 50–60 nm thickness were collected. Sections were stained in uranyl acetate and lead citrate. Imaging was done using a Technai G2 Transmission Electron Microscope (FEI company, Netherlands) with an ORIUS^TM^ SC200 CCD camera (Gatan Inc., USA), and analysis was done by a certified pathologist. Measures were taken to avoid or reduce external bias in the examined samples. The myocardial samples for treated and control mice were collected in similar manners and processed in parallel to ultrathin sections (i.e. synchronic embedding, same buffers, etc). The examination was done with the same instrumental set-up, consecutively for up to six sections in the same session, and for some samples repeated at later time points, while the type of treatment was unknown to the examiner (i.e., blinded fashion).

The size of the tissue analyzable by TEM is, due to technical constraints, in the range of 0.4–0.5 mm^2^ for one ultrathin section. The tissue piece for ultrastructural examination was selected by examining semi-thin, toluidine-blue stained sections of transversal heart slices in a light microscope. All samples showed normal morphology in light microscopy, thus the tissue chosen for TEM was randomly selected but included the inner wall of the left ventricle (i.e., most vulnerable to impaired perfusion) within a thickness of 0.4–0.5 mm. Between 15–25 myocardial cells and ca 60–100 capillary cross sections per heart were analyzed, comprising 2–3 separate ultrathin sections (grids)/sample. The width of desmosomal gaps for 20–40 randomly selected intercalated discs/sample was measured with the calibrated inbuilt software tool of the TEM. When images such as swollen mitochondria cristae or blood stasis were found in the majority of microscopy fields examined, they were reported as “general”, while if observations were less frequent than that, they were reported as “focal”.

### Immunostaining

Mice were anaesthetized by intraperitoneal injection of 2% avertin and perfused with 10 ml PBS (pH 7.4) and 10 ml 2% paraformaldehyde (PFA). Dissected hearts were put in 30% sucrose at 4°C overnight, embedded in OCT Cryomount (45830; Histolab, Askim, Sweden) and sectioned at 5 μm thickness. Cryosections were fixed in ice-cold methanol and unspecific binding was blocked by incubation in 3% BSA/PBS. The following primary antibodies and dilutions were used: anti-Ki67 (ab15580, 1:500; Abcam), anti-CD45 (553076, 1:200; BD), anti-CD68 (Serotec, MCA1957, 1:300), anti-PAD4 (ab214810, 1:100; Abcam), anti-CD31 (MA3105, 1:200; Thermo Fisher Scientific), anti-CD31 (BD557355, 1:1000; BD Biosciences), α-SMA-Cy3 (C6198, 1:200; Sigma) and anti-Gr1 (Ly-6 G/Ly-6C, 553123, 1:300; BD). The following secondary antibodies were used: Cy3-conjugated anti-rat (712–165-150, 1:1000; Jackson Immunoresearch), Alexa Fluor 555 anti-rabbit (A31572, 1:1000; Invitrogen) and Alexa Fluor 488 anti-hamster (127–545-160, 1:1000; Jackson ImmunoResearch). Sections were counterstained with Hoechst (H3570, 1:1000; Invitrogen) and mounted with Fluoromount-G (0100–01; Southern Biotech,). For proliferation studies, double stainings were performed with anti-Ki67 in combination with CD31, CD45 and α-SMA, respectively. At least 100 Ki67+ cells in representative areas in hearts from *n* = 3 PyMT+ mice were counted and scored positive or negative for the different cell type-markers. Imaging of the immunostained tissue sections was done using a Nikon Eclipse 90i microscope and the NIS Elements 3.2 software. Image analysis was performed using the Image J 1.45s software (National Institute of Health). Results are presented as average number of positive cells per 20x image field.

### Confocal 3D-imaging

PyMT+ mice, healthy littermates and PyMT+ mice treated with DNase I as described above were anaestheitized with 4% isoflurane and hearts were dissected and fixed in formalin buffer. Tissue sections from paraffin-embedded tissue (6 μm) were deparaffinized and first stained with NucGreen^TM^ Dead 488 ReadyProbes (SYTOX-green). Samples were then fixed and permeabilized with methanol for 10 min, washed three times with PBS and blocked for 15 min at room temperature with 10% goat serum in PBS. Tissue sections were then incubated overnight at 4°C with rat anti-mouse Gr1 (Ly-6 G/Ly-6C, 553123, 1:300; BD) antibody diluted 1:300 in PBS. Sections were washed three times with PBS and incubated for 1 h at room temperature with goat anti-rat Alexa-647 antibody (1:1,000 in PBS), washed three times with PBS and incubated at room temperature for 30 min with NucBlue^TM^ Live ReadyProbes (Hoechest 33342). Samples were washed three times with PBS and mounted using ProLong^TM^ Diamond Antifade Mountant. Images were acquired using 63x NA 1.40 oil objective on Zeiss LSM700 confocal microscope. Images were analyzed using Imaris software (Bitplane, Zurich, Switzerland). Tissue from *n* = 2 mice/group were used and at least three areas/tissue sample were scanned.

### Echocardiography

Echocardiography was performed on healthy (*n* = 9), PyMT+ (*n* = 6), and DNase I-treated PyMT+ (*n* = 5) mice under light isoflurane anesthesia (0.9%) for a maximum of 30 minutes using Vevo1100 ultrasound (Visual Sonics, Canada). Cardiac output, stroke volume, ejection fraction, and heart rate were quantified in parasternal short-axis view using the Cardio Set software (Visual Sonics, Canada). Left ventricle posterior wall thickness was measured using M-mode positioned at the largest diameter in parasternal short-axis view, carefully avoiding inclusion of the papillary muscle. Mitral valve flow was assessed with Pulsed wave Doppler in four-chamber view, and used to calculate maximum mitral valve flow, isovolumetric relaxation and contraction times, as well as ejection time.

### RNA expression analysis by qPCR

Mice were sacrificed by cervical dislocation and hearts immediately dissected and snap-frozen in isopentane on dry ice. The RNeasy Midi kit (75142; Qiagen) was used to isolate RNA and cDNA was generated using the iScript cDNA synthesis kit (1708891; Bio-Rad), according to instructions from the manufacturers. The KAPA SYBR FAST qPCR kit (KK4608; KAPA Biosystems) was used for the qPCR reaction. RNA expression is presented as relative to expression of hypoxanthine-guanine phosphoribosyltransferase (HPRT). Primer sequences are listed in Supplemental Table 2.

### Statistical analysis

Statistical analysis was performed using GraphPad Prism 8 (GraphPad Software Inc.). The non-parametric two-tailed Mann-Whitney analysis was used to compare two groups. The Spearman correlation test was used to detect a potential correlation between the two analyzed parameters. Error bars indicate the standard deviation and * is defined as *p* < .05, ** as *p* < .01, *** as *p* < .001, and **** as *p* < .0001.

## Results

### Neutrophil extracellular trap formation and increased coagulation in mice with mammary carcinoma

**Figure 1.**
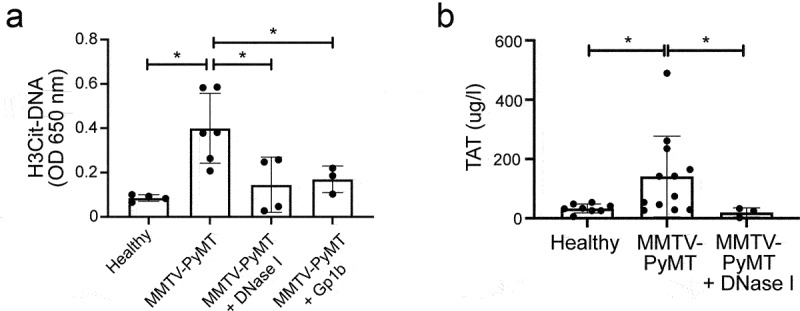
NET formation and elevated coagulation in mice with mammary carcinoma. (a) ELISA for H3Cit-DNA complexes in mouse plasma (Healthy *n* = 4; MMTV-PyMT *n* = 6; MMTV-PyMT+DNase I *n* = 4; MMTV-PyMT+Gp1b *n* = 3, Healthy vs. MMTV-PyMT *p* = .0095; MMTV-PyMT vs. MMTV-PyMT+DNase I *p* = .0381; MMTV-PyMT vs. MMTV-PyMT+Gp1b *p* = 0,0476). (b) TAT levels analyzed in mouse plasma (Healthy *n* = 8; MMTV-PyMT *n* = 12; MMTV-PyMT+DNase I *n* = 3, Healthy vs. MMTV-PyMT *p* = .0168; MMTV-PyMT vs. MMTV-PyMT+DNase I *p* = .0308). Statistical test used: non-parametric two-tailed Mann-Whitney.

In the current study, we used the spontaneous and orthotopic MMTV-PyMT (PyMT+) mouse model for mammary carcinoma.^27^ These mice uniformly develop adenocarcinomas of all mammary epithelia by 8–10 weeks of age, with a high incidence of pulmonary metastases. To address the level of NET formation in PyMT+ mice, complex formation between citrullinated histone 3 (H3Cit) and DNA was analyzed by ELISA in plasma samples. The level of H3Cit-DNA complexes was significantly increased in plasma from mice with breast cancer compared to healthy littermates (Figure 1a). Histone citrullination is performed by the enzyme peptidyl-arginine deiminase-4 (PAD4), expressed in neutrophils. PAD4 expression has also been reported in some tumors.^30^ Specificity of the H3Cit-DNA ELISA for NETs was, however, demonstrated by the exclusive expression of PAD4 by neutrophils in PyMT+ tumors (Fig. S1). Plasma levels of thrombin-antithrombin (TAT) complexes were measured to address whether PyMT+ mice display enhanced coagulation. Compared to healthy littermates, PyMT+ mice had significantly higher TAT-levels (Figure 1b), indicating a pro-thrombotic state. Treatment with DNase I daily during 3 days to degrade NETs, resulted in a reduction of the H3Cit-DNA complexes (Figure 1a), as well as a reduced level of TAT-complexes (Figure 1b).

In addition to NETs being pro-thrombotic, activated platelets have also been reported to contribute to NET formation.^31^ In agreement, platelet depletion of PyMT+ mice by administration of an anti-Gp1b antibody, reduced the level of H3Cit-DNA complex formation (Figure 1a), indicating a reciprocal relationship between NET formation and the hemostatic system in the MMTV-PyMT model.

### Mice with breast cancer display inflammation and NET-like structures in the myocardium

**Figure 2.**
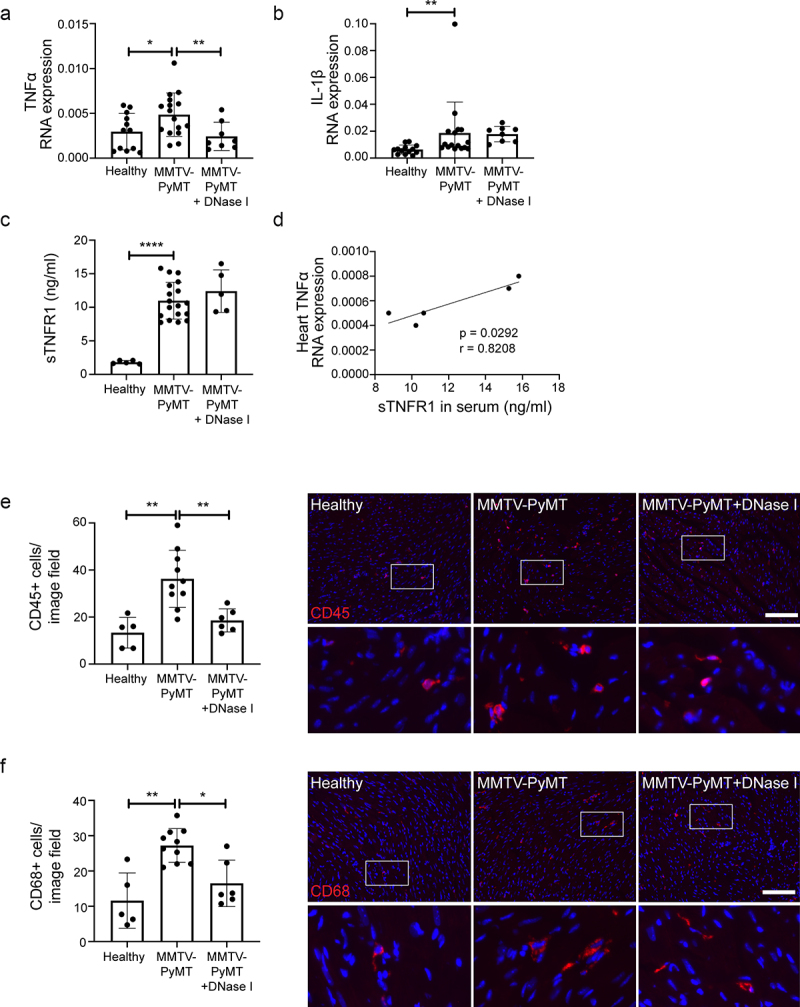
Mice with mammary carcinoma display cardiac inflammation. Heart-derived RNA was analyzed by qPCR for (a) TNFα (Healthy *n* = 12; MMTV-PyMT *n* = 16; MMTV-PyMT+DNase I *n* = 8, Healthy vs. MMTV-PyMT *p* = .0340; MMTV-PyMT vs. MMTV-PyMT+DNase I *p* = .0091) and (b) IL-1β (Healthy *n* = 13; MMTV-PyMT *n* = 16; MMTV-PyMT+DNase I *n* = 8, Healthy vs. MMTV-PyMT *p* = .0037; MMTV-PyMT vs. MMTV-PyMT+DNase I *p* = .0926). (c) ELISA for sTNFR1 in plasma (Healthy *n* = 5; MMTV-PyMT *n* = 18; MMTV-PyMT+DNase I *n* = 5, Healthy vs. MMTV-PyMT *p* < .0001; MMTV-PyMT vs. MMTV-PyMT+DNase I *p* = .3630). (d) Correlation between plasma sTNFR1 levels and RNA expression levels of TNFα in heart tissue (*n* = 5, *p* = .0292, *r* = 0.8208). RNA expression is presented as relative expression to HPRT. Heart tissue sections were immunostained for (e) CD45 (Healthy *n* = 5; MMTV-PyMT *n* = 10; MMTV-PyMT+DNase I *n* = 6, Healthy vs. MMTV-PyMT *p* = .0013; MMTV-PyMT vs. MMTV-PyMT+DNase I *p* = .0030) and (f) CD68 (Healthy *n* = 5; MMTV-PyMT *n* = 10; MMTV-PyMT+DNase I *n* = 6, Healthy vs. MMTV-PyMT *p* = .0043; MMTV-PyMT vs. MMTV-PyMT+DNase I *p *= .0146). Scale bars indicate 100 μm. Statistical test used: non-parametric two-tailed Mann-Whitney and Spearman correlation test.

Previous data from our group show that PyMT+ mice have impaired perfusion of the myocardium, paralleled with an increased number of neutrophils in the heart tissue.^19^ To examine in more detail if PyMT+ mice show signs of myocardial inflammation, we analyzed expression of inflammatory cytokines in the heart by qPCR. Expression of TNFα (Figure 2a) and IL-1β (Figure 2b) were significantly elevated in hearts from PyMT+ mice, compared to healthy littermates. Treatment with DNase I for 3 days was sufficient to suppress the elevated TNFα expression to levels seen in hearts from healthy littermates (Figure 2a), while IL-1β expression was unaffected by the treatment (Figure 2b). Shedding of TNFRs is induced in cardiomyocytes as a response to high TNFα levels, as a protection from extensive inflammation-induced signaling. Increased levels of soluble TNFR1 has also been associated with various types of heart disease.^32^ ELISA on serum showed that sTNFR1 levels were significantly increased in PyMT+ mice compared to healthy littermates (Figure 2c). Furthermore, there was a correlation between the level of sTNFR1 and the expression of TNFα in heart tissue (Figure 2d). The DNase I treatment was not sufficient to reduce the amount of sTNFR1 protein in serum from PyMT+ mice (Figure 2c).

In line with the increased expression of pro-inflammatory cytokines, there was an elevated number of CD45+ leukocytes in heart tissue from PyMT+ mice (Figure 2 e). Immunostaining for CD68 revealed that the majority of the heart-associated leukocytes were macrophages, with a significantly higher number in PyMT+ mice compared to healthy littermates (Figure 2 f). Treatment with DNase I reduced the number of CD45+ and CD68+ cells in heart tissue from mice with breast cancer (Figure 2 e and f).

As described above, we have previously shown that neutrophil infiltration is increased in hearts from PyMT+ mice compared to healthy littermates.^19^ To examine these neutrophils in more detail, we analyzed hearts from healthy and PyMT+ mice, as well as PyMT+ mice treated with DNase I, using 3D confocal microscopy. In contrast to the previous immunostainings performed on cryosections from perfused mice, we now omitted the perfusion before resection and fixated the cardiac tissue in PFA, followed by paraffin-embedding. This procedure would reduce the risk of losing intravascular NETs. Immunostaining for neutrophils (anti-Gr1) and DNA was performed and confirmed our previous findings of increased anti-Gr1 immunoreactivity in PyMT+ mice compared to healthy littermates. This high-resolution 3D analysis of the heart tissue revealed Hoechest 33342-stained fibers close to what appeared to be fragmented Gr1+ cells in hearts from PyMT+ mice. The fragmented Gr1+ cells constituted approximately 1/3 of all Gr1+ cells. These NET-like structures were not detected in hearts from either healthy or DNase I-treated PyMT+ mice (Fig. S2).

While no apparent histological alteration and no fibrosis was observed with light microscopy (data not shown), analysis by electron microscopy revealed several signs of inflammation and tissue damage in hearts from tumor-bearing mice (Fig. S3), including general blood stasis and platelet aggregation in the capillaries of PyMT+ mice (Fig. S3A). Endothelial cells had mitochondria with swollen cristae (Fig. S3B), an indicator of oxidative stress. Cardiomyocytes displayed focally broken cell membranes and the desmosomal gaps of the intercalated discs, connecting neighboring cardiomyocytes in the myocard,^33^ were widened and distorted (Fig. S3C and D). These alterations were not detected in myocardium from PyMT+ mice treated with DNase I. Interstingly, we detected fibrils of about 11 nm in diameter and “beads on a string” appearance in a PyMT+ mouse capillary lumen, closely resembling previously published TEM pictures of NETs (Fig. S3E).

### Endothelial proliferation and expression of biomarkers for myocardial strain in heart tissue from mice with breast cancer

**Figure 3.**
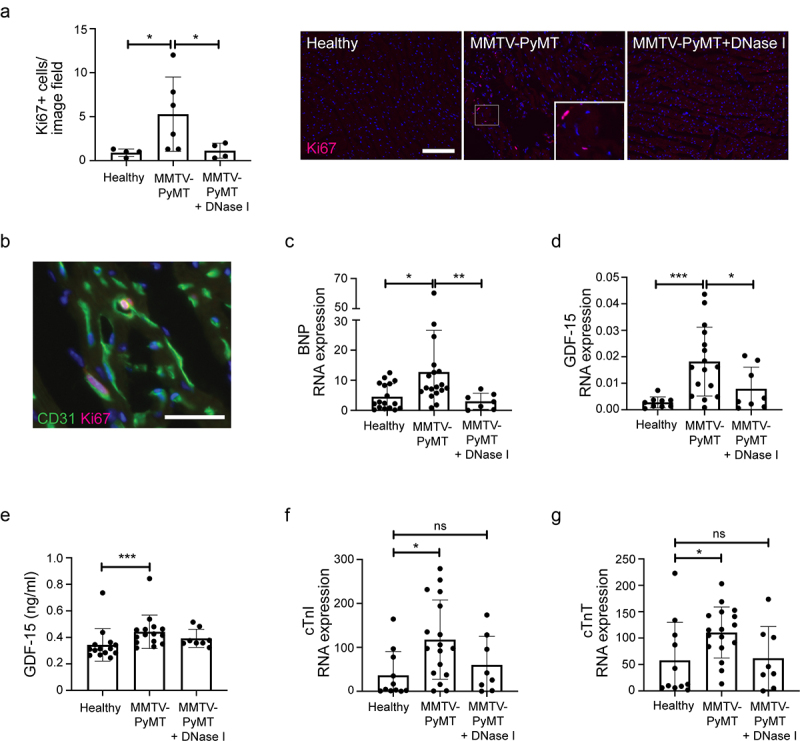
Endothelial proliferation and expression of biomarkers for myocardial strain in PyMT+ mice. (a) Proliferation of cells in heart tissue sections was analyzed by immunostaining for Ki67 (Healthy *n* = 4; MMTV-PyMT *n* = 6; MMTV-PyMT+DNase I *n* = 4, Healthy vs. MMTV-PyMT *p* = .0190; MMTV-PyMT vs. MMTV-PyMT+DNase I *p* = .0286). Scale bar indicates 100 μm. (b) Representative image showing proliferating (Ki67+) endothelial (CD31+) cells. RNA from heart tissue was analyzed by qPCR for expression of (c) BNP (Healthy *n* = 17; MMTV-PyMT *n* = 18; MMTV-PyMT+DNase I *n *= 7, Healthy vs. MMTV-PyMT *p* = .0126; MMTV-PyMT vs. MMTV-PyMT+DNase I *p* = .0023) and (d) GDF-15 (Healthy *n* = 9; MMTV-PyMT *n* = 16; MMTV-PyMT+DNase I *n* = 8, Healthy vs. MMTV-PyMT *p* = .0002; MMTV-PyMT vs. MMTV-PyMT+DNase I *p* = .0448). RNA expression is presented as relative expression to HPRT. (e) ELISA for GDF-15 in serum (Healthy *n* = 14; MMTV-PyMT *n* = 15; MMTV-PyMT+DNase I *n *= 8, Healthy vs. MMTV-PyMT *p* = .0008; MMTV-PyMT vs. MMTV-PyMT+DNase I *p* = .3833). RNA from heart tissue was analyzed by qPCR for expression of (f) cTnI (Healthy *n* = 11; MMTV-PyMT *n* = 17; MMTV-PyMT+DNase I *n* = 8, Healthy vs. MMTV-PyMT *p* = .0109; Healthy vs. MMTV-PyMT+DNase I *p* = .4421), (g) cTnT (Healthy *n* = 11; MMTV-PyMT *n* = 17; MMTV-PyMT+DNase I *n *= 8, Healthy vs. MMTV-PyMT *p* = .0168; Healthy vs. MMTV-PyMT+DNase I *p *= .8404). RNA expression is presented as relative expression to HPRT. Scale bar indicates 3 μm. Statistical test used: non-parametric two-tailed Mann-Whitney.

To address whether any remodeling of the heart can be detected in mice with breast cancer, proliferation was analyzed by immunostaining for Ki67. An increased number of proliferating cells were detected in hearts from PyMT+ mice compared to healthy littermates (Figure 3a). To identify which cell type that is proliferating, we performed co-immunostainings for Ki67 and either CD31 (endothelial cells), CD45 (leukocytes) or α-SMA (fibroblasts). Counting of least 100 Ki67+ cells in representative areas from *n* = 3 PyMT+ mice showed that the majority of the proliferating cells (77%) were also positive for the endothelial marker CD31 (see Figure 3b for representative image). Only 2% of the Ki67+ cells were CD45+ and none α-SMA+. We therefore conclude that the absolute majority of the proliferating cells in the analyzed hearts are endothelial cells. DNase I treatment suppressed proliferation to levels comparable to healthy mice (Figure 3a).

Inflammation and vascular proliferation of the heart tissue indicate that the myocardium could be damaged in PyMT+ mice. To address this further, we analyzed biomarkers associated with inflammation and myocardial strain in tissue and blood samples collected from PyMT+ mice and healthy littermates. Expression of B-type natriuretic peptide (BNP), associated with prolonged stress and elevated pressure in the myocardium and also a marker for heart failure and myocardial strain,^34,35^ was elevated in heart tissue from PyMT+ mice compared to healthy littermates (Figure 3c). Upon treatment with DNase I, the elevated expression of BNP was significantly reduced (Figure 3c). GDF-15 is a cytokine produced by cardiomyocytes as a response to inflammation and is strongly associated with major adverse cardiac events and death.^36–38^ Analysis of GDF-15 expression by qPCR revealed an upregulation in hearts derived from PyMT+ mice compared to healthy mice, which was suppressed by DNase I treatment (Figure 3d). Serum levels of GDF-15 were also significantly higher in PyMT+ mice compared to healthy littermates (Figure 3e). Treatment with DNase I for 3 days resulted in a trend toward lower serum GDF-15 levels, although not significantly different from untreated PyMT+ mice (Figure 3e). Transcriptional analysis of the cardiac troponins I and T, secreted in response to cardiac damage,^39^ revealed significantly increased expression in hearts from PyMT+ mice compared to healthy littermates (Figure 3 f and g). DNase I treatment of PyMT+ mice resulted in expression levels indistinguishable from those in healthy littermates (Figure 3 f and g), but not significantly reduced compared to hearts from untreated PyMT+ mice.

### Functional heart measurements using echocardiography

To assess if there are any differences in heart function, we performed echocardiography of healthy (*n* = 9), PyMT+ (*n* = 6), and DNase I-treated PyMT+ (*n* = 5) mice. The analyzed parameters include mitral valve flow, heart rate, isovolumic relaxation time (IVRT), end-systolic volume (ESV), end-diastolic volume (EDV), cardiac output (CO), stroke volume (SV), ejection fraction (EF) and left ventricle posterior wall thickness in diastole (LVPW;d) (Fig. S4). The results show that CO was substantially increased in PyMT+ mice because of higher SV and a trend toward higher heart rate. This was associated with increased mitral valve flow, and shortened IVRT in mice with mammary carcinoma compared to healthy littermates. In addition, there was posterior wall hypertrophy in PyMT+ mice. Taken together, these data indicate an increased work load on the heart in mice with mammary carcinoma, which at this timepoint is well-compensated. Treatment with DNase I for 3 days did not result in significant changes of these parameters.

### Patients with treatment-naïve malignancies have elevated plasma levels of inflammatory and cardiac biomarkers, which correlate with the presence of NETs

**Figure 4.**
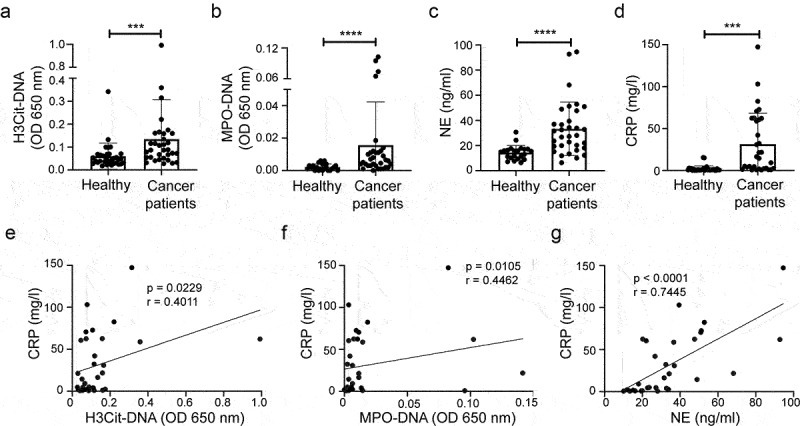
NET formation and inflammation in patients with malignancies. Plasma from patients with various types of malignant disorders were analyzed by ELISA for (a) H3Cit-DNA complexes (Healthy *n* = 33; Cancer patients *n* = 33, *p* = .0002), (b) MPO-DNA complexes (Healthy *n* = 22; Cancer patients *n* = 33, *p* < .0001) and (c) neutrophil elastase (NE) (Healthy *n* = 22; Cancer patients *n* = 33, *p* < .0001). (d) CRP concentration in plasma (Healthy *n* = 33; Cancer patients *n* = 32, *p* = .0002). (e) Correlation between H3Cit-DNA complexes and CRP in cancer patient plasma (*n* = 32, *p* = .0229, *r* = 0.1296). (f) Correlation between MPO-DNA complexes and CRP in cancer patient plasma (*n* = 32, *p* = .0105, *r* = 0.4462). (g) Correlation between NE and CRP in cancer patient plasma (*n* = 32, *p* < .0001, *r* = 0.7445). Statistical test used: non-parametric two-tailed Mann-Whitney and Spearman correlation test.

To address the translational relevance of our findings in mouse, we analyzed the correlation between NETs and biomarkers for cardiac injury in plasma samples from treatment-naïve patients with a range of different malignant disorders. NETs were measured using both the H3Cit-DNA ELISA described above and an ELISA for complex formation between myeloperoxidase (MPO) and DNA (MPO-DNA). Both NET analyses showed significantly higher levels in cancer patients than in an age- and sex-matched healthy control group (Figure 4 a and b). Neutrophil elastase (NE), another neutrophil protease and a component of NETs, was also elevated in plasma from cancer patients (Figure 4c). The H3Cit-DNA levels in the patient samples correlated well with both MPO-DNA (Fig. S5A) and NE (Fig. S5B) levels, supporting their relevance as readouts for NETs.

Our mouse data suggest that tumor-induced NETs are associated with inflammation. To address whether this is the case also in cancer patients, we measured the inflammatory marker C-reactive protein (CRP) in plasma. CRP was significantly higher in cancer patients compared to the healthy control group (Figure 4d). Furthermore, the levels of H3Cit-DNA complexes (Figure 4e), MPO-DNA complexes (Figure 4f) and NE (Figure 4g) all correlated with CRP, indicating that NETs are associated with inflammation also in cancer patients. An overall medical asessment of the patients inflammatory status from 0 to 3 (where 0 = no inflammation and 3 = severe inflammation), based on several parameters (CRP, sedimentation rate, haptoglobin, orosomucoid, alpha1-antitrypsin and plasma proteinfractions) also showed a correlation to H3Cit-DNA and NE in plasma (Fig. S6), further supporting this conclusion. The level of H3Cit-DNA was significantly higher in grade 3 patients compared to grade 0 (Fig. S6A) and NE was significantly higher in grade 3 patients compared to both grade 0 and grade 1 patients (Fig. S6C). There was, however, no significant correlation between MPO-DNA levels and inflammatory status (Fig. S6B).

**Figure 5.**
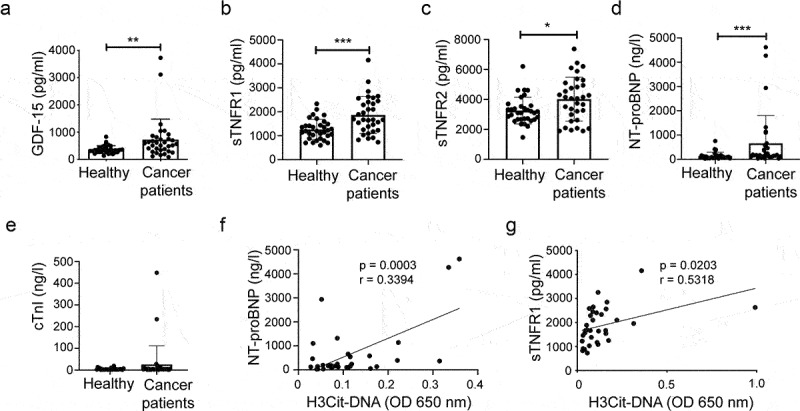
Patients with malignancies have elevated levels of biomarkers for cardiovascular disease. Plasma analysis of (a) GDF-15 (Healthy *n* = 33; Cancer patients *n* = 33, *p* = .0020), (b) sTNFR1 (Healthy *n* = 33; Cancer patients *n* = 33, *p* = .0004), (c) sTNFR2 (Healthy *n* = 33; Cancer patients *n* = 33, *p* = .0274), (d) NT-proBNP (Healthy *n* = 32; Cancer patients *n* = 32, *p* = .0004) and (e) cTnI (Healthy *n* = 33; Cancer patients *n* = 33, *p* = .0584). Analysis of correlation between (f) H3Cit-DNA and NT-proBNP (*n* = 32, *p* = .0003, *r* = 0.3394), and (g) H3Cit-DNA and sTNFR1 (*n* = 33, *p* = .0203, *r* = 0.5318). Statistical test used: non-parametric two-tailed Mann-Whitney and Spearman correlation test.

Biomarkers reflective of inflammation, myocardial strain and death were analyzed in plasma from the same cohort of treatment-naïve patients and healthy individuals. The levels of GDF-15 (Figure 5a), sTNFR1 (Figure 5b), sTNFR2 (Figure 5c) and NT-pro-BNP (Figure 5d) were all significantly increased in patients with malignant disorders compared to the healthy control group. Two cancer patients, a man with pancreatic cancer and a woman with mammary carcinoma, had pathological levels of the cardiac-specific protein troponin I in the plasma (Figure 5e). Interestingly, these two individuals also had among the highest levels of NETs, as well as of NT-proBNP (data not shown).

To determine whether NET formation was associated with biomarkers for cardiac disease in patients with malignant disorders, we performed a correlation analysis with respect to NETs and the analyzed biomarkers. There was a correlation between the level of H3Cit-DNA and the level of both NT-proBNP (Figure 5f) and sTNFR1 (Figure 5g), indicating that NET formation may indeed be connected to cardiac injury in patients with malignancies.

## Discussion

Cancer mortality is frequently caused by thrombosis and impaired organ function such as renal and heart failure, even though these organs are not sites for tumor growth. Here, we show for the first time that NETs can promote cardiac inflammation and injury in mice with breast cancer, and establish a correlation between the presence of NETs and biomarkers of cardiovascular disease in the circulation of patients with different types of malignancies. These findings suggest that NET formation may be a contributing factor to cardiac dysfunction and stress in cancer patients, independent of any therapy. Tumor-induced NETs could represent a potential therapeutic target to prevent systemic organ damage and enable optimal anti-cancer treatment.

Despite the well-known connection between malignancies and the increased risk for thrombotic events,^9,40^ the underlying mechanisms are partly unknown and appear to be multifaceted.^41^ The pro-thrombotic nature of cancer has been attributed to the expression of various pro-coagulant factors produced by the tumor cells or exposed by the dysfunctional tumor vasculature. Extracellular DNA, derived from dying tumor cells or neutrophils undergoing NET formation, can also contribute to thrombosis, since it activates the intrinsic coagulation pathway via factor XII. In recent years it has become increasingly clear that there is a close connection between coagulation and inflammation and the concept of immunothrombosis has emerged.^42^ Immunothrombosis results from the concerted action between leukocytes, platelets and coagulation factors. NETs have been shown to play an important role in this event by providing a surface where platelets can become activated, with subsequent generation of thrombin and deposition of fibrin that contributes to the formation of a clot. NET-induced immunothrombosis has been reported in preclinical models of cancer.^18,43,44^ In agreement, H3Cit levels in plasma – a biomarker for NETs – was found to predict venous thromboembolism in cancer patients.^17^ Recent studies have indeed found a correlation between NET levels and survival of cancer patients.^45–48^ Considering the involvement of NETs in organ failure and thrombosis, major causes of cancer-associated death, this is not a surprising finding. Our data show that mice with breast cancer display both enhanced NET formation and coagulation, which was reduced to normal level when NETs were dissolved using DNase I. This finding supports a causal relationship between NET formation and increased coagulation and the possibility that NETs are promoters of CAT. Interestingly, NET formation in the MMTV-PyMT model was dependent on platelets, judging by the reduced H3Cit-DNA complex formation after depletion of the platelet population. This finding is in agreement with previous studies reporting a role for platelets in NET formation^31^ and highlights the reciprocal relationship between the hemostatic system and NET formation.

Cardiac inflammation and biomarkers of myocardial strain and failure were elevated both in the MMTV-PyMT model for breast cancer and in a patient group with various malignancies, sampled before initiation of any therapy. These data are in agreement with a direct negative effect on the myocardium by cancer and not only as a side-effect of chemotherapy. For example, expression of TNFα was elevated in hearts from mice with cancer and correlated to an increasing concentration of sTNFR1 in serum. Increased levels of soluble TNF-receptors (sTNFRs) were also detected in plasma from cancer patients and has been associated with cardiovascular disease in several studies.^49^ A correlation between TNFα and H3Cit levels in plasma from cancer patients were also reported, supporting the connection between NETs and inflammation.^45^ There is in fact an intricate interplay between NETs and inflammation. Induction of NET formation is frequently reported in inflammatory conditions, such as cancer, diabetes and autoimmune disorders and several inflammatory mediators that contribute to NET formation have been identified.^13,50–52^ At the same time, NETs themselves have the capacity to cause inflammation due to their cytotoxic and procoagulant properties and have in several cases been suggested to contribute to disease progression.^14,18,53^ A challenge is therefore to define causes and consequences in these situations. It is fully possible that inflammation-induced NET formation contributes to certain aspects of a disease progression, while other aspects are independent of NET formation. Removal of NETs, for example by DNase I treatment, is therefore an important tool in these investigations.

In our study, short-term treatment with DNase I suppressed the elevated NET levels in PyMT+ mice, measured as H3Cit-DNA complex formation, as well as the increased coagulation activity, measured as TAT complexes, back to levels comparable to healthy mice. Furthermore, several of the factors that were upregulated at the RNA level in PyMT+ mice, such as TNFα, GDF-15 and BNP, were decreased to levels of healthy mice when NETs were dissolved with DNase I treatment. No effects of short-term DNase I treatment were, however, detected at the protein level. It is likely that a longer period of DNase I treatment is required to obtain a reversion of the elevated protein levels in plasma. Prolonged treatment with DNase I is, however, not possible, since no murine-derived DNase I is commercially available. The DNase I used in this study is of bovine origin and repeated injection in mice will therefore provoke an immune response against the injected protein. A previous study performed by us revealed that a majority of mice developed anti-DNase I antibodies after 1 week of daily injections of bovine DNase I^20^ and consequently any effects of the treatment will be lost after this time. Since none of the mice had detectable anti-DNase I antibodies after 3 days of treatment with bovine DNase I, we have accordingly limited the treatment duration in this study to 3 days. A potential approach to enable long-term treatment with DNase I is adeno-associated virus (AAV) based gene therapy.^54^ Such an approach would enable a continuous and endogenous supply of mouse DNase I, without the need of daily injections.

Proliferation was increased in hearts from PyMT+ mice, indicative of tissue remodeling. A recent study showed that ischemia, caused by myocardial infarction, induced proliferation of fibroblasts and endothelial cells, rather than cardiomyocytic proliferation.^55^ Our data also show that the absolute majority of the proliferating cells in PyMT+ hearts are endothelial cells. This is relevant considering previous studies showing that intravascular NETs cause damage to the endothelium.^56,57^ Confocal microscopy and 3D imaging to visualize neutrophils and DNA in non-perfused cardiac tissue (to avoid flushing out intravascular NETs) revealed NET-like structures in hearts from PyMT+ mice. These structures were not seen in cardiac tissue from healthy mice or PyMT+ mice treated with DNase I. Likewise, the increased proliferation in hearts from PyMT+ mice was suppressed by the DNase I treatment, supporting the conclusion that NETs are responsible for the increased endothelial proliferation in the myocardium of PyMT+ mice. There was also an increased expression of cardiac troponin I and T in hearts from mice with breast cancer compared to healthy controls, which should originate from the cardiomyocytes. Whether this increased transcriptional activity of cTnI and cTnT reflects a regeneration of injured cardiomyocytes is currently not known. DNase I treatment resulted in cTnI and cTnT expression levels that were indistinguishable from those in healthy littermates, but not significantly reduced compared to hearts from untreated PyMT+ mice.

No histopathological alterations or fibrosis were detected by routine light microscopy. However, electron microscopy revealed pathological changes in the endothelial cells and cardiomyocytes in hearts from mice with mammary carcinoma. Platelet aggregates and blood stasis were observed in the capillaries of PyMT+ mice. This is in agreement with previously published data from our group showing impaired perfusion of the myocardium in PyMT+ mice.^19^ Mitochondria with swollen cristae, an indicator of oxidative stress, were observed in endothelial cells of PyMT+ mice but not in healthy control mice. The intercalated disc (ID), a highly organized type of cell-cell connection along and between neighboring cardiomyocytes, were widened and distorted in PyMT+ mice. The ID is composed of desmosomes, adherens junctions and gap junctions and is essential for synchronous electrical and mechanical coupling between cardiomyocytes and, consequently, for proper myocardial function.^33^ Based on previous studies connecting disturbances of the ID to cardiomyopathies and remodeling of the myocardium,^58,59^ it can be surmised that this plays a role in the alterations in the myocardium indicated by the other analyses. PyMT+ mice treated with DNase I had focally slightly widened intercalated discs, but otherwise no differences from healthy mice, indicating a role for NETs in these histological alterations.

Echocardiography did not detect reduced function of the myocardium in PyMT+ mice compared to healthy littermates. However, increased cardiac output, myocardial hypertrophy and a trend toward higher heart rate was detected in PyMT+ mice. A possible explanation for the increased cardiac ouput could be an increased metabolic activity due to the tumor burden. Another potential explanation is reduced peripheral oxygenation. In support of the latter is our previous findings of reduced vascular perfusion of kidney and heart in PyMT+ mice compared to healthy littermates, which was reverted upon removal of NETs by DNase I treatment.^19^ Interestingly, a very recent study (published during revision of this manuscript) reports that NETs can cause immunothrombosis in the small vessels of the myocardium, leading to cardiac hypertrophy and dysfunction in a mouse model of angiotensin II-cardiomyopathy.^60^ DNase I treatment for 3 days did not affect the functional parameters measured by echocardiography in our study. It is, however, not unlikely that changes at the level of gene transcription are detected earlier and that a longer treatment period would be needed to alter functional parameters.

Analysis of patient plasma samples indicates that NETs could play a similar role in tumor-associated organ damage in humans as in mice. Inflammation, as well as NET formation, was increased in both humans and mice with malignant disorders. Cancer patients showed increased CRP levels, indicative of inflammation, and the level of CRP correlated with the amount of NETs in plasma. In addition, the overall inflammatory status of the patient correlated with the level of H3Cit-DNA complexes and NE in plasma. The patient cohort included in this study represents a wide variety of malignancies. It is remarkable that despite the heterogenous patient group, significant differences were observed with regard to both NET formation and several biomarkers for cardiac dysfunction compared to the healthy control group. This suggests that NET formation and associated organ damage occur in a broad spectrum of cancer patients, and not only in certain types of malignancies.

Targeting of NETs by systemic administration of DNase I has been applied without apparent side-effects in a number of preclinical disease models.^61^ Two DNases, DNase I and DNase I-like 3, are present in our circulation with the likely purpose to protect us from thrombosis induced by extracellular DNA, for example derived from damaged or dying cells. Genetic ablation of these two DNases in mouse resulted in accumulation of intravascular NETs and clot formation, which obstructed blood vessels and caused organ damage.^62^ In addition, DNase I is already in clinical use for the management of cystic fibrosis since decades, demonstrating its safety as a drug in this context.^63^ In cystic fibrosis patients, debris containing extracellular DNA is accumulating in the airways and DNase I in the form of an aerosol spray relieves the respiratory symptoms. Moreover, injection of DNase I in patients with the autoimmune disease lupus nephritis, where insufficient DNase I activity is believed to be a part of the disease mechanism, did not generate any safety signals.^64^ Collectively, these data indicate that systemic administration of DNase I is a safe therapeutic option to remove NETs.

We conclude that targeting of NETs could be a potential approach to prevent inflammation and possibly also organ dysfunction in patients with different types of malignancies.

## Supplementary Material

Supplemental Material

Supplemental Material
