# Supplementary material for: Neutrophil extracellular traps promote cancer-associated inflammation and myocardial stress
Source: Oncoimmunology. 2022 Mar 14;11(1):2049487. doi: 10.1080/2162402X.2022.2049487 (PMC8928831; doi:10.1080/2162402X.2022.2049487)
Supplement: Supplemental Material [file KONI_A_2049487_SM8490.pdf]

Supplemental Table 1: Tumor types represented in the patient cohort.

**Tumor type**	**Number of patients**
Carcinoma<br>*Adenocarcinoma**<br>*Neuroendocrine tumors*	20<br>*18*<br>*2*
Myeloma	6
Lymphoma	5
Sarcoma	2

* Breast 3, Lung 3, Cholangiocarcinoma 2, Pancreas 2, Prostate 1
Kidney 1, Ovary 1, Hepatocellular carcinoma 1, Colon 1, Thyroid papillary 1
Unknown primary 2.

Supplemental Table 2: Primer sequences for qPCR.

Gene	Forward primer (5´-3´)	Reverse primer (5´-3´)
ICAM-1	CCGCTACCATCACCGTGTA	CACAGGTCTCACCTCCACACT
VCAM-1	TGGGAACCTGGAACCAAGTA	CTCTGGATCCTTGGGGAAAA
E-selectin	GCGCTTTCTCTCTGCTCTTG	ATGAGCTCACTGGAGGCATT
IL1β	TGAAATGCCACCTTTTGACA	GGGTCCGTCAACTTCAAAGA
CXCL1	GTCGCGAGCCTTGCCTTCAC	AAGCCAGCGTTCACCAGACAGG
TNFα	GTCTACTGAACTTCGGGGTGA	AGGGTCTGGGCCATAGAACT
HPRT	CAAACTTTGCTTTCCCTGGT	TCGAGAGGTCCTTTTCACC
cTnI	GCCCTCAAACTTTTTCTTTCGC	CTGATGGTGCAGATTGCGAAG
cTnT	GAAGCAGGAGATGGAACGAG	TTAAACTTGCCACGGAGGTC
BNP	ATGGATCTCCTGAAGGTGCTG	GTGCTGCCTTGAGACCGA
