# Supplementary material for: Neutrophil extracellular traps promote cancer-associated inflammation and myocardial stress
Source: Oncoimmunology. 2022 Mar 14;11(1):2049487. doi: 10.1080/2162402X.2022.2049487 (PMC8928831; doi:10.1080/2162402X.2022.2049487)
Supplement: Supplemental Material [file KONI_A_2049487_SM0834.pdf]

Supplemental Figure 1

A

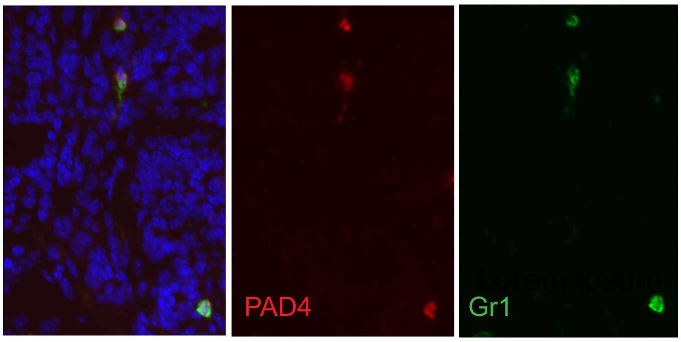

Supplemental Figure 2

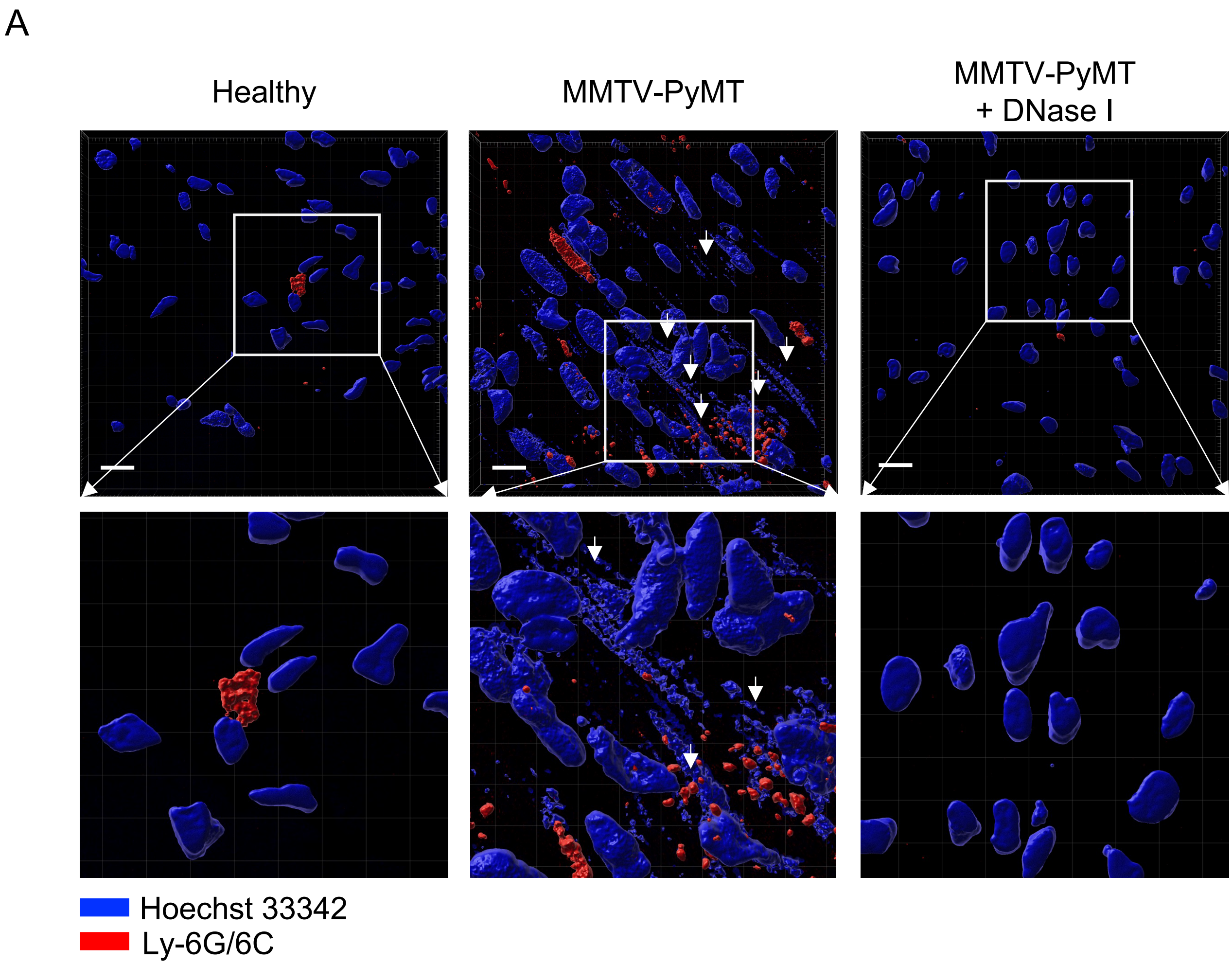

Supplemental Figure 3

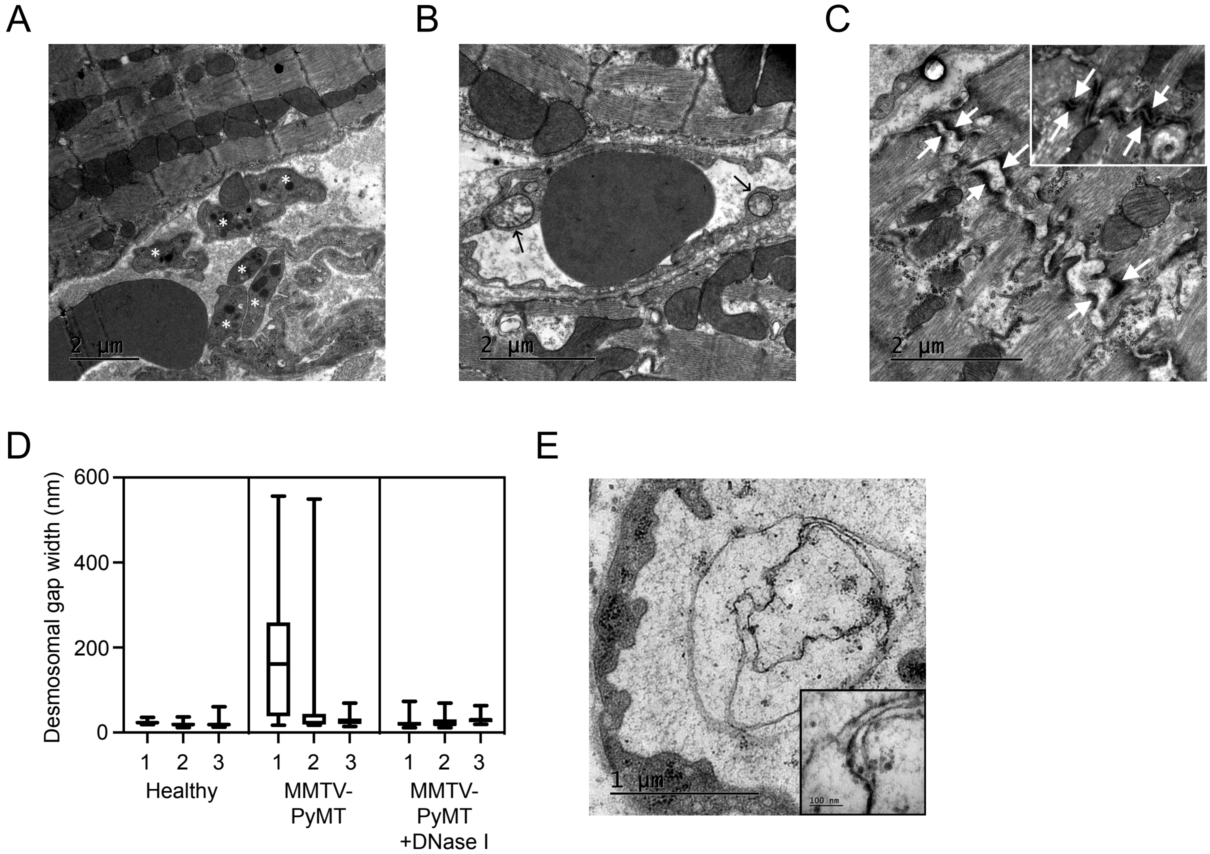

Supplemental Figure 4

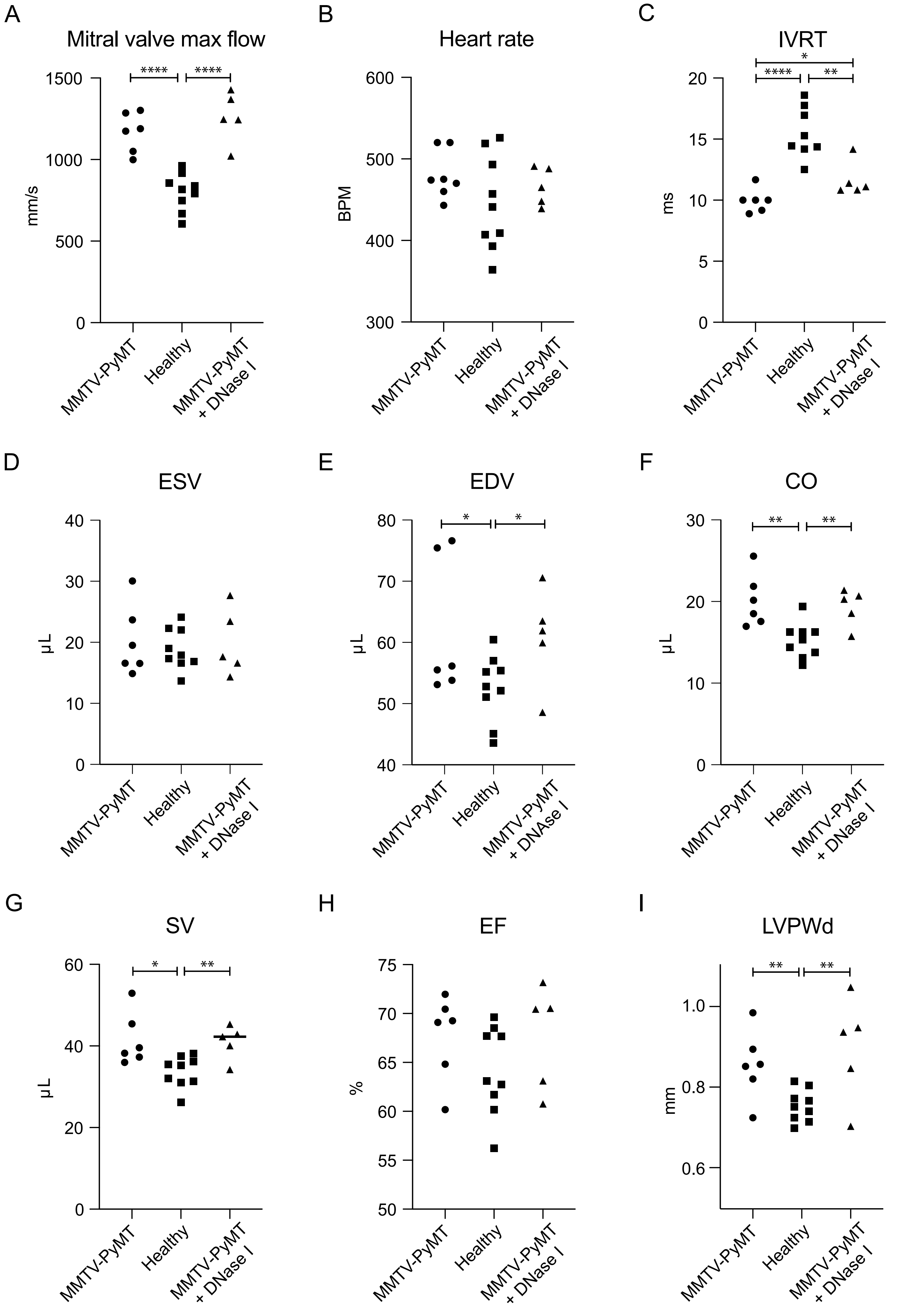

Supplemental Figure 5

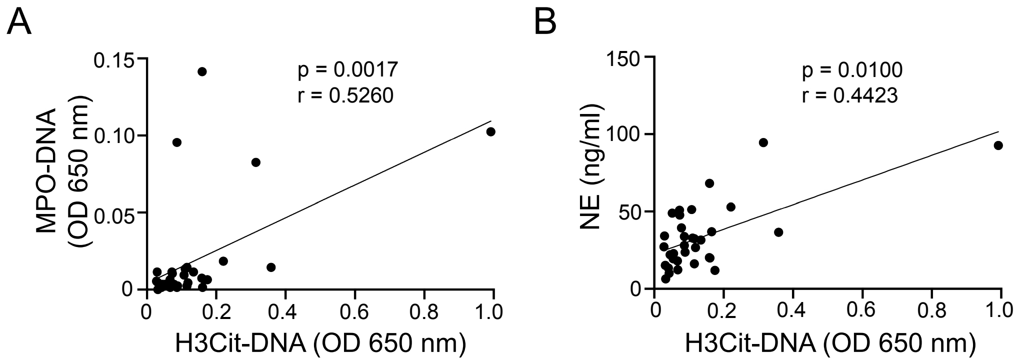

Supplemental Figure 6

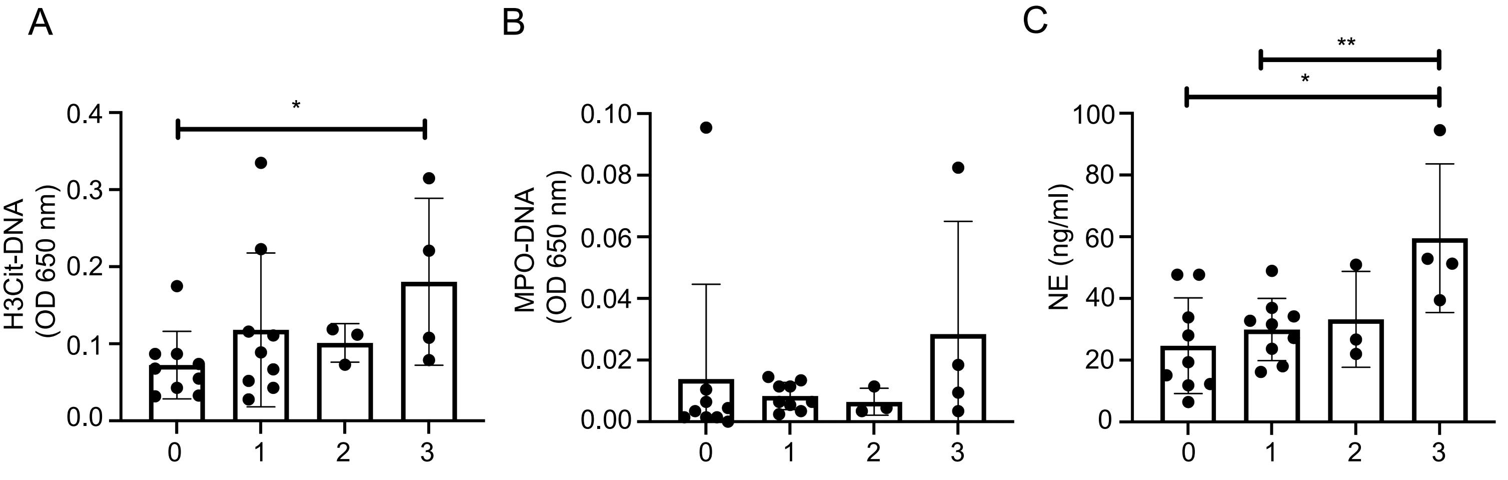

SUPPLEMENTAL FIGURE LEGENDS

Supplemental Figure 1: PAD4 is expressed specifically in neutrophils and not in MMTV-PyMT breast cancer cells.

Imunostaining for neutrophils (Gr1) and PAD4 on MMTV-PyMT tumor tissue.

Supplemental Figure 2: NET-like structures are present in cardiac tissue from PyMT+ mice.

Representative 3D confocal microscope images of cardiac tissue from healthy, PyMT+ and DNase I-treated PyMT+mice, immunostained for neutrophils (Ly6G/6C) and DNA (Hoechst 33342). White arrows indicate NET-like structures with elongated DNA fibers and fragmented neutrophils in hearts from PyMT+ mice. Scale bars indicate 10 μm.

Supplemental Figure 3: Inflammation and tissue damage in the myocard from PyMT+ mice.

Analysis by electron microscopy revealed (A) general blood stasis in the capillaries with aggregates of platelets (*) and (B) mitochondria with swollen cristae (black arrows) in the endothelial cells but no abnormalities of the basal lamina, in heart tissue from PyMT+ mice. (C) Cardiomyocytes from PyMT+ mice had widened and distorted intercalated discs (indicated by pair of arrows) and focally broken cell membranes. Inset: normal intercalated disc from a healthy control. (D) A quantitative analysis of the width between the pair of desmosomal dense plaques, which are an important component of the intercalated disc, revealed a remarkable widening especially in one individual with cancer, for which the width between the pair of desmosomal dense plaques was in many cases 10 times larger than normal (ca 200 nm versus the normal 19-23 nm). Notably, this mouse had the highest tumor burden

of the three PyMT+ mice included in the analysis (data not shown). Another PyMT+ mouse displayed a more moderat width increase, while a third individual was not significantly different from the healthy control mice. PyMT+ mice treated with DNase I had focally slightly widened intercalated discs, but otherwise no differences from healthy mice. Each bar represent measurements from one individual mouse. (E) A capillary lumen from a PyMT+ mouse with fibrils of about 11 nm in diameter and “beads on a string” appearance, closely resembling previously published TEM pictures of NETs *in vitro* [1, 2]

Supplemental Figure 4: Echocardiography on healthy, PyMT+and DNase I-treated PyMT+ mice.

Cardiac function was assessed by echocardiography on healthy (n=9), PyMT+ (n=6), and DNase I-treated PyMT+ (n=5) mice. The analysed papameters include mitral valve flow, heart rate, isovolumic relaxation time (IVRT), end-systolic volume (ESV), end-diastolic volume (EDV), cardiac output (CO), stroke volume (SV), ejection fraction (EF) and left ventricle posterior wall thickness in diastole (LVPW;d).

Supplemental Figure 5: Correlation between NET assays and NE in plasma from patients with malignant disorders.

Plasma from patients with various types of malignant disorders were analyzed by ELISA for NETs using two different assays, either H3Cit-DNA or MPO-DNA. (A) Correlation between H3Cit-DNA and MPO-DNA (n=33, p=0.0017, r=0.5260). (B) Correlation between H3Cit-DNA and neutrophil elastase (NE) (n=33, p=0.0100, r=0.4423).

Supplemental Figure 6: Correlation between patients inflammatory status and NETs. An overall medical asessment of the patients inflammatory status from 0-3 (where 0 = no inflammation and 3 = severe inflammation), based on several parameters (CRP, sedimentation rate, haptoglobin, orosomucoid, alpha1-antitrypsin and plasma proteinfractions) was performed for each patient. (A) The level of H3Cit-DNA in plasma was significantly higher in grade 3 patients compared to grade 0. (B) The level of MPO-DNA in plasma did not show a significant correlation to inflammatory status. (C) The level of NE in plasma was significantly higher in grade 3 patients compared to grade 0 and grade 1.

[1] Pilsczek, F. H., Salina, D., Poon, K. K., Fahey, C., Yipp, B. G., Sibley, C. D., Robbins, S. M., Green, F. H., Surette, M. G., Sugai, M., Bowden, M. G., Hussain, M., Zhang, K., and Kubes, P. (2010) A novel mechanism of rapid nuclear neutrophil extracellular trap formation in response to Staphylococcus aureus. *J Immunol* **185**, 7413-7425

[2] Richmond, T. J., Finch, J. T., Rushton, B., Rhodes, D., and Klug, A. (1984) Structure of the nucleosome core particle at 7 A resolution. *Nature* **311**, 532-537
